# Cancer on Hold, Infections on the Rise: The Unseen Pandemic Effect on Thoracic Surgery in the Developing World

**DOI:** 10.7759/cureus.82623

**Published:** 2025-04-20

**Authors:** Muhammad Imran, Elham Shakil, Shehryar Khan, Hira Bakhtiar Khan, Maha Wazir, Farhan Ullah, Dawood Tahir, Muhammad Moeed, Qaidar Alizai, Imran Tahir

**Affiliations:** 1 Department of Thoracic Surgery, Lady Reading Hospital Medical Teaching Institution, Peshawar, PAK; 2 Department of Thoracic Surgery, Hayatabad Medical Complex Peshawar, Peshawar, PAK

**Keywords:** cancer, covid-19, oncology, surgery, thoracic

## Abstract

Background

The COVID-19 pandemic disrupted global healthcare systems, leading to major shifts in surgical case prioritization. This study examines how thoracic surgical trends changed at a tertiary care hospital in Peshawar, Pakistan, specifically comparing oncologic and non-oncologic procedures, patient demographics, and surgical outcomes.

Methods

We conducted a retrospective comparative study of major thoracic surgeries performed between December 2018 and July 2021. The study period was divided into pre-pandemic (December 2018-March 2020) and pandemic (April 2020-July 2021) phases. Surgical records were reviewed to assess changes in oncologic and non-oncologic surgical indications, patient characteristics, and outcomes. Univariate analyses were conducted to compare the pre-pandemic and pandemic groups for baseline characteristics, indications, and procedures. Multivariable analyses identified the independent association of the pandemic with oncologic procedures. Data analysis was performed using IBM SPSS Statistics for Windows, Version 25 (Released 2017; IBM Corp., Armonk, New York, United States).

Results

A total of 246 thoracic surgeries were analyzed. Oncologic surgeries significantly declined from 36 (41.4%) pre-pandemic to 34 (21.4%) during the pandemic (p=0.001), with esophagectomy for esophageal carcinoma decreasing from 16 (18.4%) to 9 (5.7%) (p<0.002) and lobectomy for lung cancer dropping from six (6.9%) to one (0.6%) (p=0.018). Meanwhile, non-oncologic surgeries increased significantly from 51 (58.6%) to 125 (78.6%) during the pandemic (p<0.001). The most notable increases were in decortication for empyema thoracis, from 21 (24.1%) to 55 (34.6%), p=0.006, and emergency thoracotomy, from two (2.3%) to 11 (6.9%), p=0.034. Hydatid cystectomy remained stable from 11 (12.6%) to 17 (10.7%), p=0.722. While baseline patient characteristics remained similar, thoracic surgeries were performed more frequently in males and children under 10 years old (p=0.042) during the pandemic. Despite the increased volume, postoperative complications (p=0.39) and mortality rates (p=0.553) remained unchanged.

Conclusion

The COVID-19 pandemic significantly altered thoracic surgical trends, shifting the focus from oncologic to non-oncologic cases, particularly infectious and emergency conditions. The decline in cancer surgeries reflects disruptions in oncologic care, while the rise in empyema thoracis highlights increased infectious disease burden and late-stage presentations. These findings emphasize the need for strategies that ensure continued oncologic care while responding to emergent thoracic conditions during global health crises.

## Introduction

In late 2019 and early 2020, a novel coronavirus emerged in China and rapidly evolved into a global pandemic [1,2,3]. The virus was initially called 2019-nCoV, later designated SARS-CoV-2, and the disease caused by this virus was named COVID-19 [2]. Despite strict containment measures, including travel restrictions, quarantines, and hygiene protocols, the virus rapidly spread globally, leading to widespread healthcare disruptions. While COVID-19 primarily affected the respiratory system, it also contributed to secondary complications such as bacterial pneumonia, thromboembolic events, and delayed care for non-COVID conditions due to the burden on the healthcare systems [3,4,5].

Beyond its direct medical impact, COVID-19 drastically affected surgical procedures, shifting priorities from elective and oncologic surgeries to emergency and infection-related procedures. Many hospitals around the world faced severe crises with surgical triaging and care, but the effects seen were different in developed and developing countries. Unlike the first-world countries, infections are not as huge a problem as in the third-world countries, where most of our patients present with acute, severe, and advanced infections. It has been shown that during the pandemic, immediately life-threatening non-oncologic conditions, such as empyema thoracis and trauma, were prioritized over the care of slow-growing oncologic conditions [6,7]. While this was necessary to address the immediately life-threatening conditions, the shift resulted in delayed cancer diagnoses, treatment interruptions, and a decline in oncologic thoracic surgeries. The extent of disruption varied based on the healthcare capacity of the area, resource allocation, and crisis response strategies [8].

In Pakistan, where healthcare infrastructure is already underdeveloped, pandemic-related delays in the care of cancer patients may have led to disease progression and worse long-term outcomes for cancer patients. Conversely, an increased burden of non-oncologic thoracic conditions, particularly bacterial and parasitic infections, likely reflected the indirect results of the pandemic [9]. While similar patterns were observed globally, few studies have quantified these changes in thoracic surgical practice in developing countries.

This study aims to assess the effect of the COVID-19 pandemic on the covariates and outcomes of oncologic and non-oncologic thoracic surgeries at a tertiary care hospital in Pakistan [10,11]. We analyzed the before and after trends in surgical case volume, patient demographics, and postoperative outcomes with the spread of the virus. This study will provide deep insights into how the pandemic affected surgical prioritization and will give important information on the implications of these findings for any future crisis in a resource-limited country.

## Materials and methods

Study setting

This study was conducted at the Hayatabad Medical Complex (HMC), which is a large 1,300-bed tertiary care hospital in Peshawar, a city in the north of Pakistan. This hospital receives a large number of patients from across the Khyber Pakhtunkhwa Province and neighboring Afghanistan. The Thoracic Surgery Unit, established in 2018, provides outpatient, elective, and emergency services. With an enormous patient burden and limited resources, hospitals faced significant challenges in both surgical and medical management during the COVID-19 pandemic. These challenges make it an ideal setting to analyze the effects of the pandemic on patient care.

Study design and data collection

This retrospective, comparative descriptive study analyzed all major thoracic surgical cases performed at our hospital between December 1, 2018, and July 31, 2021. After approval from the Institutional Review Board (IRB), we manually extracted data from the operation theater (OT) records using a continuous probability sampling technique.

Inclusion and exclusion criteria

All patients undergoing major thoracic procedures under general anesthesia during the study were included. Data was collected from the OT records using the convenience sampling technique. Patients with incomplete records, such as missing demographic or procedural data, and those undergoing procedures performed under local anesthesia were excluded due to inconsistent documentation.

Stratification

While COVID-19 originated in 2019, it only spread in Pakistan after March 2020, leading to the first nationwide lockdown on April 1, 2020 [10,11]. Based on this timeline, the study period was divided into two 16-month periods: a pre-pandemic phase (December 1, 2018, to March 31, 2020) and a pandemic phase (April 1, 2020, to July 31, 2021).

Data was further categorized into two groups based on surgical indications: oncologic thoracic surgeries, such as esophagectomy for esophageal cancer, lobectomy for lung cancer, mediastinal tumor resection, and biopsies; non-oncologic thoracic surgeries, such as decortication for empyema thoracis, hydatid cystectomy, emergency thoracotomy, and feeding jejunostomy.

Data points

Relevant patient and surgical details were recorded for each case, including baseline characteristics (age and gender), surgical indication, type of procedure performed, elective vs. emergency status, and postoperative outcomes such as complications and in-hospital mortality.

Statistical analysis

Data was entered into IBM SPSS Statistics for Windows, Version 25 (Released 2017; IBM Corp., Armonk, New York, United States) for analysis. Descriptive and comparative statistical methods were applied to assess trends before and during the pandemic. An independent sample t-test was used to compare continuous variables, such as patient age, while Pearson’s chi-square test and Fisher’s exact test were applied to categorical variables, including gender distribution, surgical indication, procedure, complications, and mortality. Continuous variables were reported as means and standard deviation (SD), whereas categorical variables were presented as frequencies and percentages. A p-value<0.05 was considered statistically significant.

## Results

A total of 246 major thoracic surgeries were performed, with a 53% increase in surgical volume from 87 cases pre-pandemic to 159 during the pandemic.

Baseline characteristics

The mean age of patients decreased significantly from 37.79 years (pre-pandemic) to 31.19 years (pandemic), p=0.017. A significant increase in surgeries among children (<10 years old) was observed (10.7% to 21.5%, p=0.042), whereas the proportion of older patients (>50 years) decreased from 31% to 20.3%. No statistically significant differences were found in gender distribution (64% male, 35% female, p=1.000) or in the elective vs. emergency status (p=0.811) (Table 1).

**Table 1:** Comparison of the baseline characteristics of patients who underwent any major thoracic surgery before and during the COVID-19 pandemic. Percentages may not total 100 based on rounding; statistically significant p-values are written in bold

Variable	Total (n=246)	Pre-pandemic (n=87)	Pandemic (n=159)	p-value	Test Statistic (Test Used)
Age (Mean ± SD, years)	33.49 ± 18.29	37.79 ± 18.34	31.19 ± 17.81	0.017	t=2.41 (Independent t-test)
Age Group Distribution					
- Children (<10 years), n (%)	44 (17.9%)	10 (10.7%)	34 (21.5%)	0.042	χ²=4.13 (Chi-square test)
- Young (11-50 years), n (%)	143 (58.1%)	50 (58.3%)	93 (58.3%)	0.999	χ²=0.00 (Chi-square test)
- Older Adults (>50 years), n (%)	59 (24.0%)	27 (31.0%)	32 (20.3%)	0.06	χ²=3.55 (Chi-square test)
Female Gender, n (%)	87 (35.4%)	31 (35.6%)	56 (35.2%)	1.000	χ²=0.00 (Fisher's exact test)
Elective Cases, n (%)	225 (91.5%)	79 (90.5%)	146 (91.8%)	0.811	χ²=0.06 (Chi-square test)
Emergency Cases, n (%)	21 (8.5%)	8 (9.5%)	13 (8.2%)	0.811	χ²=0.06 (Chi-square test)

Among oncologic cases, the mean age was similar between the pre-pandemic (52.6 ± 15.2 years) and pandemic (54.1 ± 14.8 years) periods (p=0.65), with no significant differences in gender distribution (30.6% vs. 29.4% female, p=0.91) or age groups (66.7% vs. 70.6% older adults, p=0.72). While there was no statistically significant difference in the mean age of the pre-pandemic and pandemic groups among the non-oncologic patient group, the mean age of this group overall was significantly lower than the oncologic group (non-oncologic group: 52.6 (15.2) years vs. 28.3 (16.4) years). Gender distribution remained stable (39.2% vs. 36.8% female, p=0.76) in the two time periods (Tables 2, 3).

**Table 2:** Comparison of baseline characteristics of oncologic cases before and during the pandemic.

Variable	Total n=70	Pre-pandemic n=36	Pandemic n=34	p-value	Test Value (Test Statistic)
Age (Mean ± SD, years)	53.3 ± 14.9	52.6 ± 15.2	54.1 ± 14.8	0.65	t=-0.46 (t-test)
Female Gender, f(%)	21 (30.0%)	11 (30.6%)	10 (29.4%)	0.91	χ²=0.01 (Chi-square)
Children (<10 years), f(%)	0 (0%)	0 (0%)	0 (0%)	-	N/A
Young (11-50 years), f(%)	22 (31.4%)	12 (33.3%)	10 (29.4%)	0.72	χ²=0.13 (Chi-square)
Older Adults (>50 years), f(%)	48 (68.6%)	24 (66.7%)	24 (70.6%)	0.72	χ²=0.13 (Chi-square)

**Table 3:** Comparison of baseline characteristics of non-oncologic cases before and during the pandemic.

Variable	Total n=176	Pre-pandemic n=51	Pandemic n=125	p-value	Test Value (Test Used)
Age (Mean ± SD, years)	26.5 ± 16.1	28.3 ± 16.4	25.7 ± 15.9	0.32	t=0.99 (t-test)
Female Gender, f(%)	66 (37.5%)	20 (39.2%)	46 (36.8%)	0.76	χ²=0.09 (Chi-square)
Children (<10 years), f(%)	44 (25.0%)	10 (19.6%)	34 (27.2%)	0.29	χ²=1.11 (Chi-square)
Young (11-50 years), f(%)	121 (68.8%)	38 (74.5%)	83 (66.4%)	0.30	χ²=1.07 (Chi-square)
Older Adults (>50 years), f(%)	11 (6.2%)	3 (5.9%)	8 (6.4%)	0.90	p=1.0 (Fisher’s exact test)

Oncologic vs. non-oncologic surgery trends

A significant decline in oncologic thoracic surgeries was observed, decreasing from 41.4% pre-pandemic to 21.4% during the pandemic (p=0.001). The most affected oncologic procedures included esophagectomy for esophageal carcinoma (25.3% to 9.4%, p<0.001) and lobectomy for lung cancer (6.9% to 0.6%, p=0.018). In contrast, non-oncologic thoracic surgeries increased significantly (p<0.001), accounting for 78.6% of cases during the pandemic. The most notable increase was seen in empyema thoracis (27.6% to 40.3%, p=0.005), which became the most common indication for thoracic surgery during the pandemic (Tables 4, 5). The decline in oncologic procedures likely reflects disruptions in elective cancer care and diagnostic delays, whereas the rise in empyema cases may be attributed to delayed medical care, secondary bacterial infections, and post-COVID-19 pulmonary complications.

**Table 4:** Comparison of the oncologic indications for major thoracic surgeries before and during the COVID-19 pandemic. Percentages may not total 100 based on rounding; a: percentages are calculated out of the total procedures done (both oncologic and non-oncologic); Fisher's exact test is used for small sample sizes (expected cell count <5); statistically significant p-values are written in bold; f (%): frequency (percentage)

Variable, f(%) ^a^	Total (n=246)	Pre-Pandemic (n=87)	Pandemic (n=159)	p-value	Test Statistic (Test Used)
All Oncologic Indications	70 (28.5%)	36 (41.4%)	34 (21.4%)	0.001	χ²=11.3 (Chi-square)
Esophageal Carcinoma	37 (15.0%)	22 (25.3%)	15 (9.4%)	<0.001	χ²=12.6 (Chi-square)
Lung Cancer/Lung Mass	7 (2.8%)	6 (6.9%)	1 (0.6%)	0.02	p=0.02 (Fisher's exact test)
Mediastinal Tumor	6 (2.4%)	1 (1.1%)	5 (3.1%)	0.17	p=0.17 (Fisher's exact test)
Others (Stomach Cancer, etc.)	20 (8.1%)	7 (8.0%)	13 (8.2%)	0.92	χ²=0.01 (Chi-square)

**Table 5:** Comparison of the non-oncologic indications for major thoracic surgeries before and during the COVID-19 pandemic. Percentages may not total 100 based on rounding; a: percentages are calculated out of the total procedures done (both oncologic and non-oncologic); b: p-value could not be calculated when any one of the cell values was zero; Fisher's exact test is used for small sample sizes (expected cell count <5); statistically significant p-values are written in bold; f (%): frequency (percentage) Trauma (e.g., firearm injury, road traffic accident, stab wound, crush injury); Chest Wall Conditions (e.g., abscess, sinus, hematoma, benign tumors); Esophageal Injuries (e.g., corrosive injury, post-laparotomy gastroesophageal junction injury); Others (e.g., diaphragmatic hernia, bronchiectasis, mediastinal cyst, pleural conditions, emphysema, mycetoma)

Variable, f (%) ^a^	Total (n=246)	Pre-Pandemic (n=87)	Pandemic (n=159)	p-value	Test Statistic (Test Used)
All Non-oncologic Indications	176 (71.5%)	51 (58.6%)	125 (78.6%)	<0.001	χ²=12.9 (Chi-square)
Empyema Thoracis	88 (35.8%)	24 (27.6%)	64 (40.3%)	0.005	χ²=7.9 (Chi-square)
Hydatid Cyst	28 (11.4%)	11 (12.6%)	17 (10.7%)	0.72	χ²=0.13 (Chi-square)
Trauma	17 (6.9%)	4 (4.6%)	13 (8.2%)	0.28	p=0.96 (Fisher's exact test)
Esophageal Injuries	3 (1.2%)	2 (2.3%)	1 (0.6%)	0.34	p=0.34 (Fisher's exact test)
Chest Wall Conditions	9 (3.7%)	0 (0%)	9 (5.7%)	N/A ^b^	N/A ^b^
Pneumothorax/Recurrent Lung	7 (2.8%)	0 (0%)	7 (4.4%)	N/A ^b^	N/A ^b^
Others	23 (9.3%)	10 (11.5%)	13 (8.2%)	0.46	χ²=0.55 (Chi-square)

Surgical procedures

Pre-pandemic, the most frequently performed procedures were decortication (24.1%), esophagectomy (18.4%), and hydatid cystectomy (12.6%). During the pandemic, a significant increase in decortication procedures (24.1% to 34.6%, p=0.006) was observed, including both open and video-assisted thoracoscopic surgery (VATS) approaches. In contrast, esophagectomies declined sharply (18.4% to 5.7%, p=0.002), mirroring the reduction in oncologic surgical indications. The frequency of emergency thoracotomies increased (2.3% to 6.9%, p=0.034), likely reflecting prioritization of urgent and life-threatening conditions over elective cancer surgeries (Tables 6, 7).

**Table 6:** Comparison of the frequency and proportions of major thoracic surgical procedures for oncologic indications before and during the pandemic. Percentages may not total 100 based on rounding; a: percentages are calculated out of the total procedures done (both oncologic and non-oncologic); b: p-value could not be calculated when any one of the cell values was zero; Fisher's exact test is used for small sample sizes (expected cell count <5); statistically significant p-values are written in bold; f (%): frequency (percentage)

Variable, f (%) ^a^	Total (n=246)	Pre-Pandemic (n=87)	Pandemic (n=159)	p-value	Test Statistic (Test Used)
All Oncologic Procedures	70 (28.5%)	36 (41.4%)	34 (21.4%)	0.001	χ²=11.3 (Chi-square)
Esophagectomy	25 (10.2%)	16 (18.4%)	9 (5.7%)	0.002	χ²=9.6 (Chi-square)
Lobectomy	7 (2.8%)	6 (6.9%)	1 (0.6%)	0.018	p=0.018 (Fisher's exact test)
Mediastinal Mass Resection	6 (2.4%)	1 (1.1%)	5 (3.1%)	0.415	p=0.41 (Fisher's exact test)
Pleural Mass Excision	5 (2.0%)	1 (1.1%)	4 (2.5%)	0.655	p=0.65 (Fisher's exact test)
Chest Wall Tumor Excision	3 (1.2%)	0 (0%)	3 (1.9%)	0.552	N/A ^b^
Lung Biopsy for Malignancy	5 (2.0%)	1 (1.1%)	4 (2.5%)	0.655	p=0.65 (Fisher's exact test)
Mediastinal Biopsy	4 (1.6%)	0 (0%)	4 (2.5%)	0.303	N/A ^b^
Pneumonectomy	1 (0.4%)	0 (0%)	1 (0.6%)	1.000	N/A ^b^
Other Oncologic Procedures	18 (7.3%)	11 (12.6%)	7 (4.4%)	0.028	χ²=4.8 (Chi-square)

**Table 7:** Comparison of the frequency and proportions of major thoracic surgical procedures for non-oncologic indications before and during the pandemic. Percentages may not total 100 based on rounding; a: percentages are calculated out of the total procedures done (both oncologic and non-oncologic); b: p-value could not be calculated when any one of the cell values was zero; statistically significant p-values are written in bold; f (%): frequency (percentage) VATS: video-assisted thoracoscopic surgery; GI: gastrointestinal Chest Wall Procedures (e.g., abscess drainage, sinus excision, reconstruction); Pneumothorax Procedures (e.g., pleurectomy, wedge resection, bullectomy, Claggett window); Esophageal & Gastrointestinal (e.g., feeding jejunostomy, hiatal hernia repair, diaphragm repair); Others (e.g., sternotomy, thoracostomy under GA, re-exploration, hematoma evacuation, lung biopsy for benign disease, etc.)

Variable, f (%) ^a^	Total (n=246)	Pre-Pandemic (n=87)	Pandemic (n=159)	p-value	Test Statistic (Test Used)
All Non-oncologic Procedures	176 (71.5%)	51 (58.6%)	125 (78.6%)	<0.001	χ²=12.9 (Chi-square)
Decortication (Open/VATS)	76 (30.9%)	21 (24.1%)	55 (34.6%)	0.006	χ²=7.6 (Chi-square)
Hydatid Cystectomy	28 (11.4%)	11 (12.6%)	17 (10.7%)	0.722	χ²=0.13 (Chi-square)
Emergency Thoracotomy	13 (5.3%)	2 (2.3%)	11 (6.9%)	0.034	p=0.034 (Fisher's exact test)
Chest Wall Procedures	5 (2.0%)	0 (0%)	5 (3.1%)	0.042	N/A ^b^
Pneumothorax Procedures	9 (3.7%)	0 (0%)	9 (5.7%)	0.044	N/A ^b^
Esophageal and GI Procedures	17 (6.9%)	10 (11.5%)	7 (4.4%)	0.028	χ²=4.8 (Chi-square)
Other Non-oncologic Procedures	28 (11.4%)	7 (8.0%)	18 (11.3%)	0.280	χ²=1.2 (Chi-square)

Postoperative outcomes

No statistically significant differences were found in the in-hospital mortality rates (0% pre-pandemic vs. 1.9% during the pandemic) or postoperative complications (3.6% vs. 7.0%, p=0.39). While these findings suggest that surgical safety was maintained despite increased case volume, the rise in complications, though not statistically significant, may indicate higher surgical acuity and delayed presentations of non-oncologic conditions (Table 8).

**Table 8:** Comparison of post-operative outcomes before and during the pandemic for both the oncologic and non-oncologic cases. Percentages may not total 100 based on rounding; f(%): frequency (percentage)

Postoperative Outcomes (Overall)	Total (n=246)	Pre-pandemic (n=87)	Pandemic (n=159)	p-value	Test Value (Test Used)
- In-Hospital Mortality, n (%)	3 (1.2%)	0 (0.0%)	3 (1.9%)	0.553	χ²=0.35 (Fisher's exact test)
- Complications, n (%)	14 (5.7%)	3 (3.6%)	11 (7.0%)	0.390	χ²=0.74 (Chi-square test)
Oncologic Cases	Total (n=70)	Pre-pandemic (n=36)	Pandemic (n=34)		
Complications	6 (8.6%)	2 (5.6%)	4 (11.8%)	0.42	p=0.42 (Fisher's exact test)
Mortality	0 (0%)	0 (0%)	0 (0%)	-	N/A
Non-oncologic Cases	Total (n=176)	Pre-pandemic (n=51)	Pandemic (n=125)		
Complications	8 (4.5%)	1 (2.0%)	7 (5.6%)	0.44	p=0.44 (Fisher's exact test)
Mortality	3 (1.7%)	0 (0%)	3 (2.4%)	0.55	p=0.55 (Fisher's exact test)

## Discussion

This study found a clear shift in the trends of thoracic surgeries and their indications during the COVID-19 pandemic. While the overall number of surgeries increased, there was a noticeable decline in oncologic procedures and a rise in procedures for non-oncologic indications. The increase in the number of surgeries might be related to the gradual development of our thoracic surgery division, which was established in 2018 and has since been growing both in resources and services. Surgeries for esophageal cancer and lung malignancies became less frequent, while procedures for conditions like empyema thoracis and trauma-related emergencies became more frequent. Despite these obvious changes seen with the pandemic, the surgical outcomes remained stable, which might suggest a steady standard of care maintained by the hospital despite the challenges of the pandemic.

Interestingly, our findings contrast with reports from other regions, where thoracic surgical trends varied significantly. A study from Italy reported an increase in esophageal cancer resections from 21.7% to 40% [12], while a French study reported a rise in overall cancer surgeries from 65.9% to 85.6% [13]. The authors attributed these shifts in numbers to the suspension of elective procedures and a relatively low overall infection rate in those populations, which consequently prioritized the care of oncologic patients. However, in our study, oncologic surgeries, particularly for esophageal carcinoma, declined sharply during the pandemic. One likely explanation is that the incidence of esophageal cancer, among others, has been increasing in the Afghan population in the last few decades, who then seek care in bordering cities like Peshawar [14,15]. With travel restrictions and the closure of the Afghanistan-Pakistan border, the pandemic might have contributed to a direct delay in cancer diagnoses and treatment with a reduction in oncologic surgeries.

At the same time, non-oncologic cases surged, with empyema thoracis emerging as the most common indication for thoracic surgery. Both open and VATS decortications became more frequent, reflecting a higher burden of complicated pleural infections. A study from China reported no major change in the rates of empyema [9]; our experience was different. The downsizing or closure of other cardiothoracic units likely diverted more patients to our center, increasing the surgical burden for us. Additionally, delayed diagnoses (secondary to travel restrictions), worsening socioeconomic conditions, nutritional deficiencies, and immunosuppression during the pandemic may have predisposed individuals to severe infections, which also contributed to the increased incidence of empyema [16,17]. These findings highlight how disruptions in healthcare systems can lead to a rise in preventable surgical conditions when primary care and infection control services are compromised.

Unlike empyema, hydatid cyst surgeries remained stable rather than increasing significantly (p=0.72). Considering that hydatid disease is endemic in our region [18], disruptions in early healthcare access again might have delayed diagnosis, leading to complicated hydatid cysts with a higher proportion of patients requiring surgical intervention in the latter half of the pandemic. These findings emphasize the importance of maintaining the necessary screening and treatment programs for endemic diseases, even during global crises.

Despite the increased surgical volume, we saw no substantial increase in the complication and mortality rates. This suggests that the thoracic surgical teams effectively adapted to new challenges. However, an unexpected trend was the significant increase in surgeries among children under 10 years of age (p=0.042). This could reflect changes in disease presentation, delay in seeking healthcare attention, or increased severity of pediatric conditions during the pandemic. Further research is advised to determine whether these trends indicate a true shift in disease patterns or are a result of limited healthcare access.

As with all retrospective studies, this research has certain limitations. Surgical decisions are influenced by multiple factors, including surgical expertise, comorbid conditions of the patients, disease severity, access to healthcare, and resource availability, all of which could have contributed to the observed trends. Additionally, this was a single-center, small-sample study in a hospital in Pakistan, limiting the generalizability of our findings. Being a retrospective study, this study might have any or all the limitations of a retrospective study, like unknown confounders, missing data, improper categorization, recall bias, non-response bias, selection bias, and observation bias. Future multicenter studies or meta-analyses could provide a broader perspective on the impact of the pandemic on thoracic surgery. Further research should focus on quantifying the long-term consequences of surgical delays, particularly for oncologic conditions, and investigating the reasons behind the increased pediatric surgical burden. Finally, this was a retrospective, quantitative, data-based study with no assessment of qualitative patient and surgeon factors. Focusing on this aspect will provide clearer indicators of improvements.

## Conclusions

The distribution of thoracic surgeries was significantly affected during the COVID-19 pandemic, with a noticeable shift from procedures for oncologic to non-oncologic indications. Though the total number of cases of non-oncologic conditions increased, the proportion of surgeries for lung and esophageal cancer declined. The ability of surgical teams to quickly adapt to evolving barriers is demonstrated by the steady surgical results, including mortality and complication rates, in spite of these changes. These findings highlight the importance of maintaining access to oncologic care in times of crisis, strengthening infection control measures to prevent severe respiratory infections, and ensuring flexible resource distribution to support, which are all essential to effective surgical services. Further research may use our study as a baseline for constructing future guidelines for the care of these patients.
